# Is Adjuvant Therapy Necessary for Stage IB Gastric Cancer: A Retrospective Cohort Study

**DOI:** 10.1245/s10434-024-16444-w

**Published:** 2024-11-07

**Authors:** Mingyu Gu, Binghe Zhao, Changda Sui, Minghai Wen, Xinxin Wang

**Affiliations:** Department of General Surgery, The First Medical Center of Chinese, PLA General Hospital, Beijing, China

**Keywords:** Adjuvant therapy, Stage IB gastric cancer, Prognosis factor, Recurrence, Disease free survival

## Abstract

**Background:**

The benefit of adjuvant therapy for patients with IB gastric cancer (GC) is a topic of debate. This study aimed to evaluate the benefit of adjuvant therapy for patients with IB GC.

**Methods:**

Overall, the study selected 510 IB GC patients after gastrectomy at the First Medical Center of the Chinese PLA General Hospital, Beijing, China between 2005 and 2018. Overall survival (OS) and disease-free survival (DFS) were analyzed using the Kaplan-Meier method and the log-rank test. Cox regression analyses were used to confirm the independent prognostic factors.

**Results:**

Patients who received postoperative adjuvant therapy had a longer 5-year OS (92.9 %) than those who received surgery alone (86.7 %; *P* < 0.05), but the 5-year DFS did not differ significantly between the two groups (92.6 vs. 95.0 %; *P* > 0.05). Moreover, DFS did not differ between monotherapy, and combination therapy. Uni- and multivariate analyses showed that older age was a significant risk factor for tumor recurrence. Subgroup analyses also failed to identify suitable candidates for chemotherapy.

**Conclusions:**

Because adjuvant therapy did not demonstrate any benefits in terms of tumor recurrence or DFS, these treatment strategies may be unnecessary for IB GC patients after gastrectomy. Further studies are required to identify subgroups of IB GC patients who may benefit from adjuvant treatments.

**Supplementary Information:**

The online version contains supplementary material available at 10.1245/s10434-024-16444-w.

Gastric cancer (GC) is the fourth most common cause of cancer-related death, with GC diagnosed for more than 1 million people each year.^1,2^ Thanks to the development and general application of upper gastrointestinal endoscopic techniques and strategies, GC is diagnosed at an early stage for more and more patients. Mass screening programs and surveys have shown that stage I GC has increased by up to 50 % in Japan and South Korea during the past two decades.^3^

According to the eighth edition of the American Joint Committee on Cancer (AJCC) tumor-node-metastasis (TNM) staging system for GC, stage IB GC includes pT1N1M0 and pT2N0M0. As defined, pT1N1 GC is early GC with mucosal (T1a) or submucosal (T1b) infiltration involving one or two metastatic lymph nodes, and pT2N0 GC is tumor invasion of the muscularis propria without lymph node metastasis.^4^

Although curative surgery alone has yielded a very good survival rate for stage IB patients, 9.0–12.8 % of patients may still experience recurrence after surgery.^5,6^ Unfortunately, once recurrence occurs, the prognosis is poor, with a mean survival shorter than 1 year.^7^

Postoperative adjuvant therapy is recommended for stages II and III GC patients to reduce the risk of recurrence and improve prognosis based on several randomized controlled trials (RCTs). However, these trials included only a few IB GC patients, and the role of adjuvant therapy in stage IB GC was not reported in these trials.^8,9^ Based on the National Comprehensive Cancer Network (NCCN), both pT1N1M0 and pT2N0M0 patients with poorly differentiated or high-grade cancer, lymphovascular invasion, and neural invasion or age younger than 50 years are candidates for adjuvant therapy.^10^ The European Society of Medical Oncology (ESMO) recommends postoperative adjuvant chemotherapy for patients with stage IB GC who have not received preoperative chemotherapy.^11^ However, both Japanese and Korean guidelines recommend postoperative observation only for patients with stage IB GC.^12,13^ Therefore, the necessity of adjuvant therapy after surgery for stage IB GC remains controversial.

Consequently, we performed a retrospective analysis to investigate the survival benefits of postoperative chemotherapy for IB GC patients and attempted to identify the appropriate candidates for adjuvant chemotherapy among these patients.

## Materials and methods

### Patients

**Fig. 1:**
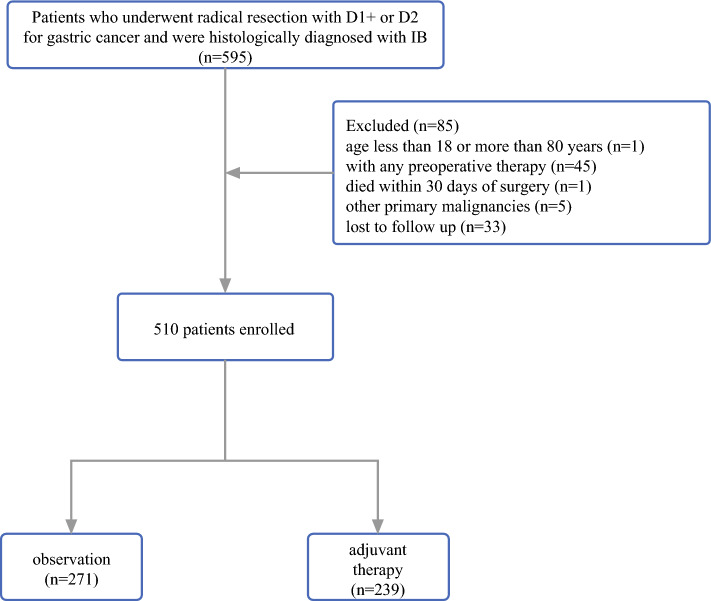
Patient selection algorithm

The study reviewed patients who underwent radical resection with D1+ or D2 lymphadenectomy for primary GC and had a histologic diagnosis of T2N0M0 or T1N1M0 gastric adenocarcinoma based on the eighth edition of the AJCC TNM staging system for GC at the First Medical Center of the Chinese PLA General Hospital, Beijing, China between 2005 and 2018. All patients were of Asian ethnicity. For this analysis, the study excluded patients younger than 18 years or older than 80 years who had undergone any preoperative therapy, had died within 30 days after surgery, had other primary malignancies, or were lost to follow-up evaluation. Finally, the study enrolled 510 patients (Fig. 1).

This study was reviewed and approved by the Ethics Committee of Chinese PLA General Hospital. (IRB file no. S2024-128-01). Due to the study’s retrospective design, the requirement for informed consent was waived.

### Patient Characteristics and Clinical Data

Clinical characteristics of age, sex, smoking, drinking, body mass index (BMI), and hospital length of stay were analyzed. Factors associated with surgical techniques included the method of approach (open, laparoscopic, or robotic), type of gastrectomy, and duration of operation. Pathologic features included for analysis in the current study were tumor location, tumor size, histologic type, lymphatic invasion, venous invasion, perineural invasion, number of retrieved lymph nodes, and positive lymph nodes.

Histologic types were reviewed according to the World Health Organization (WHO) classification and categorized into two groups according to differentiated type.^14^ Two independent, experienced pathologists reviewed hematoxylin-eosin (H&E)-stained slides from each case. If the diagnosis of the two pathologists was inconsistent, a third pathologist was needed.

### Adjuvant Therapy Strategies and Follow-up Evaluation

Because no established treatment strategy exists for IB GC, the choice of treatment depends on the preferences of the patients or doctors. Among 239 patients who received adjuvant chemotherapy, 41 (17.2 %) received single-agent S-1, 80 (33.5 %) received S-1 and oxaliplatin (SOX), 35(14.6 %) received capecitabine and oxaliplatin (CapOx), 22 (9.2 %) received fluorouracil, folinic acid, and oxaliplatin (FOLFOX), 45(18.8 %) received 5-fluorouracil and paclitaxel/docetaxel, and 16 (6.7 %) had no available details of the agents (Table S1). Effective chemotherapy was defined as receipt of at least one cycle of adjuvant chemotherapy.

The patients were evaluated every 3 or 6 months until 2 years postoperatively, then every 6 months until 5 years postoperatively. The evaluation included physical examination, hematologic examination, computed tomography, and endoscopy. Follow-up evaluation continued for at least 5 years after surgery or until censoring or death.

### Statistical Analysis

Categorical variables were compared using chi-square or Fisher’s exact tests, and continuous variables were compared using analysis of variance (ANOVA) or Kruskal–Wallis tests. Cox’s proportional hazard model was used to perform uni- and multivariate analyses. Disease-free survival (DFS) was defined as the time from surgical resection to local recurrence. Overall survival (OS) was defined as the period between surgery and the occurrence of death. The DFS and OS curves were calculated using the Kaplan-Meier method and compared by the log-rank test.

The statistical analyses were conducted using R (The R Foundation; http://www.R-project.org) and Free Statistics software version 1.8. Two-tailed tests were performed, and a *P* value lower than 0.05 was used to determine statistical significance.

Previous research indicates that the estimated 5-year OS rate for patients undergoing postoperative observation is 80 %, whereas an estimated 5-year OS rate of 90 % is demonstrated by those receiving adjuvant chemotherapy. It is anticipated that a 10 % loss to follow-up evaluation will be observed. Based on these assumptions, a sample of 213 patients per group will achieve 90 % statistical power to detect a hazard ratio (HR) of 0.472 at a significance level (alpha) of 0.15 using a two-sided test.

## Results

### Clinicopathologic Characteristics

Of the 510 patients with a diagnosis of stage IB GC, 271(53.1 %) received only observation, whereas 239 (46.9 %) received adjuvant therapy after surgery. Among the latter, 121 (23.8 %) patients were in the monotherapy group, and 118 (23.1 %) were in the combination therapy group. The ages ranged from 26 to 80 years, with a mean age of 59.1 years. The median tumor size was 2.5 cm. Age and size were converted to categorical variables, and the cutoff value was the median value.

The patients receiving adjuvant chemotherapy were younger (129 [54 %] vs 122 [45 %]; *P*<0.05) and more frequently had pT1N1 (68 [28.5 %] vs 27 [10.0 %]; *P* < 0.001). For the remaining characteristics, the two groups did not differ significantly. The baseline characteristics of the patients are detailed in Table 1.

**Table 1:** Comparison of clinicopathologic characteristics between the adjuvant chemotherapy group and the surgery-only group of patients with stage IB gastric cancer

Characteristic	Patients,			*P* Value
	Total (*n* = 510) *n* (%)	Observation (*n* = 271) *n* (%)	Adjuvant chemotherapy (*n* = 239) *n* (%)	
Sex				0.333
Female	112 (22.0)	55 (20.3)	57 (23.8)	
Male	398 (78.0)	216 (79.7)	182 (76.2)	
Mean age (years)	59.1 ± 10.3	59.9 ± 9.9	58.3 ± 10.7	0.079
Age category (years)				0.043
<60	251 (49.2)	122 (45)	129 (54)	
≥60	259 (50.8)	149 (55)	110 (46)	
Mean BMI (kg/m^2^)	23.9 ± 4.0	23.9 ± 4.2	23.9 ± 3.7	0.889
Smoke				0.895
No	310 (60.8)	164 (60.5)	146 (61.1)	
Yes	200 (39.2)	107 (39.5)	93 (38.9)	
Drink				0.633
No	315 (61.8)	170 (62.7)	145 (60.7)	
Yes	195 (38.2)	101 (37.3)	94 (39.3)	
Primary tumor location				0.079
Upper	108 (21.2)	60 (22.2)	48 (20.1)	
Medium	120 (23.6)	73 (27)	47 (19.7)	
Lower	252 (49.5)	126 (46.7)	126 (52.7)	
Overlapping	29 (5.7)	11 (4.1)	18 (7.5)	
Operation				0.946
Open	302 (59.6)	159 (58.9)	143 (60.3)	
Laparoscopy	179 (35.3)	97 (35.9)	82 (34.6)	
Robotic	26 (5.1)	14 (5.2)	12 (5.1)	
Type of gastrectomy				0.183
Distal	318 (62.4)	160 (59)	158 (66.1)	
Proximal	107 (21.0)	59 (21.8)	48 (20.1)	
Total	85 (16.7)	52 (19.2)	33 (13.8)	
TNM stage				< 0.001
T1N1	95 (18.6)	27 (10)	68 (28.5)	
T2N0	415 (81.4)	244 (90)	171 (71.5)	
Tumor embolism				0.845
No	479 (93.9)	254 (93.7)	225 (94.1)	
Yes	31 (6.1)	17 (6.3)	14 (5.9)	
Nerve invision				0.313
No	488 (95.7)	257 (94.8)	231 (96.7)	
Yes	22 (4.3)	14 (5.2)	8 (3.3)	
Lymphovascular invasion				0.763
No	36 (7.1)	20 (7.4)	16 (6.7)	
Yes	474 (92.9)	251 (92.6)	223 (93.3)	
Examined lymph nodes				0.451
<15	112 (22.0)	56 (20.7)	56 (23.4)	
≥15	398 (78.0)	215 (79.3)	183 (76.6)	
Positive lymph nodes				< 0.001
0	415 (81.4)	244 (90)	171 (71.5)	
1	56 (11.0)	13 (4.8)	43 (18)	
2	39 (7.6)	14 (5.2)	25 (10.5)	
Differentination				0.341
Differentiated	293 (57.5)	161 (59.4)	132 (55.2)	
Undifferentiated	217 (42.5)	110 (40.6)	107 (44.8)	
Median tumor size: cm (IQR)	2.5 (2.0–4.0)	2.7 (2.0–4.0)	2.5 (1.9–4.0)	0.406
Tumor size, category (cm)				0.421
<2.5	187 (36.7)	95 (35.1)	92 (38.5)	
≥2.5	323 (63.3)	176 (64.9)	147 (61.5)	
Median hospital length of stay: days (IQR)	10.0 (9.0–13.0)	10.0 (9.0–13.0)	10.0 (9.0–13.0)	0.494
Median duration of surgery: min (IQR)	199.9 (169.2–245.5)	199.7 (169.6–242.8)	200.0 (167.8–245.8)	0.817
Median blood loss: ml (IQR)	200.0 (100.0–200.0)	200.0 (100.0–200.0)	150.0 (100.0–200.0)	0.307

BMI, body mass index; TNM, tumor-node-metastasis; IQR, interquartile range

### Effect of Adjuvant Therapy

**Fig. 2:**
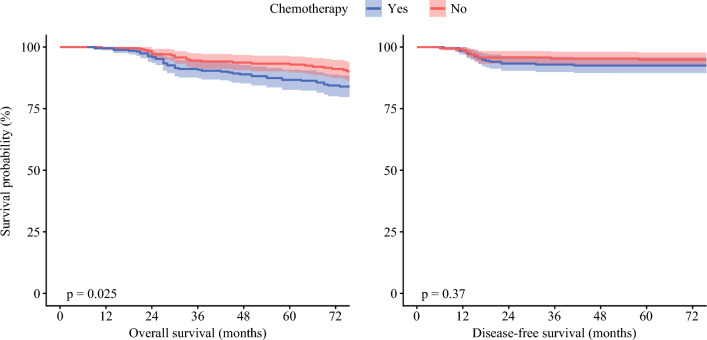
Kaplan–Meier curve for overall survival and disease-free survival between chemotherapy group and observation group

The median follow-up duration was 65.3 months. The survival analysis indicated that the patients who received postoperative adjuvant therapy had a higher 5-year OS rate (92.9 %) than those who received surgery alone (86.7 %) (*P* < 0.05; Fig. 2). However, the 5-year DFS did not differ significantly between the two groups (92.6 vs. 95.0 %; *P* > 0.05; Fig. 2).

Cox regression analyses were performed to evaluate the effect of adjuvant therapy on OS and DFS, with the surgery-only group as the reference. The HR in the uncorrected model (model 1) was 0.65 for OS (95 % confidence interval [CI]:0.31–1.78; *P*<0.05) and 0.74 for DFS (95 % CI 0.37–1.45; *P* = 0.373). After adjustment for clinical characteristics of age, sex, BMI, smoking, and drinking (model 2), the HR was 0.70 for OS (95 % CI 0.48–1.03; *P* = 0.074) and 0.77 for DFS (95 % CI 0.39–1.52; *P* = 0.451). After adjustment for clinical characteristics and surgery-related characteristics including tumor location, operation method, type of gastrectomy, duration of surgery, blood loss, and hospital length of stay (model 3), the HR was 0.71 for OS (95 % CI 0.48–1.05; *P* = 0.084) and 0.76 for DFS (95 % CI 0.38–1.52; *P* = 0.442). Finally, when all the factors were included in the analyses as covariates (model 4), the HR was 0.69 for OS (95 % CI 0.46–1.03; *P* = 0.072) and 0.61 for DFS (95 % CI 0.29–1.28; *P* = 0.191) (Table 2).

**Table 2:** Uni- and multivariable analyses of overall survival and disease-free survival in IB gastric cancer patients

Model	Model 1^a^		Model 2^b^		Model 3^c^		Model 4^d^	
	HR (95 % CI)	*P* Value	HR (95 % CI)	*P* Value	HR (95 % CI)	*P* Value	HR (95 % CI)	*P* Value
OS	0.65 (0.44–0.95)	0.026	0.7 (0.48–1.03)	0.074	0.71 (0.48–1.05)	0.084	0.69 (0.46–1.03)	0.072
DFS	0.74 (0.37–1.45)	0.373	0.77 (0.39–1.52)	0.451	0.76 (0.38–1.52)	0.442	0.61 (0.29–1.28)	0.191

HR, hazard ratio; CI, confidence interval; OS, overall survival; DFS, disease-free survival

^a^Unadjusted

^b^Adjusted for age, sex, body mass index (BMI), smoking, and drinking

^c^Model 2 + tumor location, operation method, type of gastrectomy, duration of surgery, blood loss, and hospital length of stay

^d^Adjusted for all the factors

We then compared the OS and DFS rates among the surgery-only group, the single-agent group, and the combination chemotherapy group. Compared with the surgery-only group, the HRs for OS and DFS in the single-agent therapy group were respectively 0.77 (95 % CI 0.49–1.21; *P* = 0.259) and 0.83 (95 % CI 0.37–1.88; *P* = 0.662). The HRs for OS and DFS in the combination therapy group were respectively 0.52 (95 % CI 0.31–0.89; *P* = 0.016) and 0.63 (95 % CI 0.26–1.57; *P* = 0.327). The difference between the results of monotherapy and combination chemotherapy was not statistically significant (HR for OS, 0.68 [95 % CI 0.37–1.25; *P* = 0.213] vs HR for DFS, 0.76 [95 % CI 0.26–2.19; *P* = 0.614]; Fig. S1).

To select candidates for chemotherapy, Cox regression and subgroup analyses of OS were performed with the T1N1 and T2N0 patients separately. The Cox analyses showed that neither group derived any benefit from adjuvant therapy in any of the four models (Tables S2 and S3). In the analysis of the subgroups, the patients were grouped according to sex, age, tumor size, lymphatic invasion, venous invasion, perineural invasion, differentiation, and number of lymph nodes examined. Covariates such as smoking, drinking, BMI, tumor location, hospital length of stay, and blood loss also were taken into account. The OS rates between the patients who received adjuvant chemotherapy and those who did not differ significantly in any of the subgroups (Figs. S2 and S3).

### Risk Factors of Tumor Recurrence

Of the 510 patients, 35 (6.9 %) experienced recurrence, with a recurrence rate of 7.7 % in the surgery-only group and 5.9 % in the adjuvant therapy group. The two groups did not differ statistically (Table S4). To identify the risk factors associated with recurrence, we performed uni- and multivariate Cox analyses of DFS. In the univariate analyses, only older age was found to be associated with tumor recurrence (HR, 2.2; 95 % CI 1.08–4.48; *P* = 0.02). None of the other variables were found to be associated with tumor recurrence. In the multivariate Cox regression analysis, older age remained the only independent risk factor for recurrence (HR, 2.36; 95 % CI 1.10–5.05; *P* = 0.027).

## Discussion

In recent years, due to the increasing proportion of stage IB GC, several researchers have focused on the clinical question of whether postoperative adjuvant therapy is necessary for stage IB GC.^3^ However, no unanimous conclusion has been reached.

Several studies based on public databases support the idea of postoperative adjuvant therapy. Xie et al.^15^ analyzed 470 stage IB patients from the Surveillance, Epidemiology, and End Results (SEER) database after performing a 1:1 propensity-matching score and found that the patients receiving adjuvant therapy had a more favorable prognosis. The following subgroup analyses indicated that only patients with more than 16 lymph nodes examined and a stage of pT1N1M0 were candidates for chemotherapy.

Similarly, based on the SEER database, other studies also concluded that postoperative adjuvant therapy could benefit patients with IB GC.^16–18^ In addition to SEER, another study enrolled 14,228 patients with stage IB from the National Cancer Database (NCDB) and found that adjuvant therapy had a survival benefit for the patients.^19^ Although all the studies mentioned support postoperative chemotherapy for patients with stage IB disease, it is worth noting that the 5-year survival rate for the patients ranged from 60 to 80 %.

Seoul National University Hospital in Korea reported that the 5-year OS rate for patients with stage IB GC can reach 87.6–90.2 %.^5^ The patients in our study had 5-year OS rates ranging from 82.7 to 92.9 % and DFS rates ranging from 92.6 to 95.0 %. A retrospective study conducted at Samsung Medical Centre in Korea included 738 patients with pT1N1 GC, all of whom underwent D1+ or D2 lymph node dissection and reported 5-year DFS rates ranging from 95.5 to 96.5 %.^20^ The latest multicenter study also reported a good prognosis of more than 90 % cancer-specific survival (CSS) for patients with stage IB disease.^21^ These Asian studies all reported an excellent prognosis and reached the same conclusion as we did in our study, supporting the Japanese guidelines.

The varying views on the extent of lymph node dissection in Eastern and Western countries may explain the differences in the prognoses of patients with stage IB GC. Japanese investigators have often emphasized the value of extensive lymphadenectomy (≥D2) and have considered D2 lymph node dissection for standard gastrectomy resection. According to the Japanese Gastric Cancer Association (JGCA), a D2 lymphadenectomy is indicated for cN + or ≥cT2 tumors and a D1 or D1 + for cT1N0 tumors. Because pre- and intraoperative diagnoses of tumor invasion depth and nodal involvement often are unreliable, D2 lymphadenectomy should be performed when nodal involvement cannot be excluded.^22^ However, previous Western research has not found a survival advantage when extensive lymphadenectomy is compared with a D1 resection.^23,24^ In Western medical practice, D2 dissection was once recommended but not required until Eu-90003 trials confirmed the necessity of D2.^10,25^ Consequently, previous Asian studies and our study had a much higher proportion of patients who had more than 15 lymph nodes examined (50–70 %) than Western studies (about 30 %).^15,17,18,20,21,26^

Removing as many potentially metastatic lymph nodes as possible has resulted in a better prognosis and reduced the benefit of postoperative chemotherapy for patients with more than 15 or less than 15 examined lymph nodes.^27^ This also explains why patients with examined lymph node counts lower than 15 did not benefit from adjuvant therapy and were not at high risk for recurrence in the Asian research.^20,21,26^

The postoperative chemotherapy regimens of patients with stage IB GC differ between the Chinese Society of Clinical Oncology (CSCO) clinical guidelines and NCCN. As in our study, S-1 can be used as a first-line treatment for patients with stage IB disease. In contrast, S-1 is not recommended for Western patients. On the one hand, whereas 1-year administration of S-1 for pStage II GC showed good clinical results (3-year DFS rate [93.1 %] vs 3-year OS rate [96.1 %]) in the Japan Clinical Oncology Group (JCOG) 1104 trial, no data are available for S-1 in Western patients.^28^ On the other hand, S-1 plus docetaxel is superior to S-1, with few safety concerns for patients with stage III GC. However, S-1 plus docetaxel and capecitabine plus oxaliplatin have not been directly compared among combination regimens, and thus, it remains unclear which of these combination therapies is more effective at this writing.^29,30^

It is important to select an appropriate regimen and maintain an appropriate dose and schedule, taking into consideration not only the pathologic findings, but also the patient’s general condition and the occurrence of adverse events. Further clinical trials are required to explore strategies related to the use of S-1 in combination with other chemotherapeutic agents.^31^ Consequently, S-1 has not been used in the treatment of Western patients with GC.

According to NCCN, IB GC patients with high-risk factors, including lymphovascular invasion, neural invasion, poorly differentiated high-grade cancer, and age younger than 50 years, should receive combination therapy. However, the results of our study did not support this idea, and subgroup analysis did not show that groups with high-risk factors can benefit from adjuvant therapy. Findings have shown lymph node invasion to be an independent risk factor for lymph node metastasis because tumor cells spread to lymph nodes and distant organs mainly through blood and lymphatic vasculature.^32–34^

Given the low recurrence rate of stage IB GC, analysis of high-risk factors for recurrence requires a large sample size. Therefore, our study and previous studies might have had insufficient statistical power because of an insufficient sample size to accurately identify high-risk factors for recurrence of stage IB GC.^20,35^

This study had several potential limitations. Because this was a single-center study with a relatively small number of patients with IB GC, the resulting effects, particularly in the subgroup analyses, may be underestimated, and the results should be interpreted with caution. Furthermore, it is important to note that this study was retrospective, which may have led to imbalances in baseline patient and tumor characteristics between the two groups. Finally due to the small size of the population receiving adjuvant therapy and the inconsistency of adjuvant chemotherapy regimens and treatment cycles between patients, we were unable to further subdivide the population and determine which regimen and how many cycles would be beneficial for patients with stage IB GC.

## Conclusion

Our study found no oncologic benefit of adjuvant therapy in terms of tumor recurrence or DFS for patients with IB GC. Additionally, we were unable to identify risk factors for tumor recurrence. Therefore, future retrospective studies with larger samples or even RCTs are needed to explore this clinical question and identify potential patient subgroups, as well as the most appropriate treatment option

## Supplementary Information

Below is the link to the electronic supplementary material.

Supplementary file 1 (DOCX 12 KB)

Supplementary file 2 (DOCX 13 KB)

Supplementary file 3 (DOCX 13 KB)

Supplementary file 4 (DOCX 12 KB)

Supplementary file 5 (TIF 14184 KB)

Supplementary file 6 (TIF 37190 KB)

Supplementary file 7 (TIF 36114 KB)
