# Supplementary material for: Is Adjuvant Therapy Necessary for Stage IB Gastric Cancer: A Retrospective Cohort Study
Source: Ann Surg Oncol. 2024 Nov 7;32(2):1210–7. doi: 10.1245/s10434-024-16444-w (PMC11698797; doi:10.1245/s10434-024-16444-w)
Supplement: Supplementary file 1 — (DOCX 12 KB) [file 10434_2024_16444_MOESM1_ESM.docx]

| Regimen | Number of Patients | Proportion |
| --- | --- | --- |
| Single-agent S-1 | 41 | 17.2% |
| S-1 and oxaliplatin (SOX) | 80 | 33.5% |
| Capecitabine and oxaliplatin (CapOx) | 35 | 14.6% |
| Fluorouracil, folinic acid, and oxaliplatin (FOLFOX) | 22 | 9.2% |
| Fluorouracil and paclitaxel/docetaxel | 45 | 18.8% |
| No details of agents | 16 | 6.7% |

Supplementary Table S1. Details of the Chemotherapy Regimens
