# Supplementary material for: Is Adjuvant Therapy Necessary for Stage IB Gastric Cancer: A Retrospective Cohort Study
Source: Ann Surg Oncol. 2024 Nov 7;32(2):1210–7. doi: 10.1245/s10434-024-16444-w (PMC11698797; doi:10.1245/s10434-024-16444-w)
Supplement: Supplementary file 2 — (DOCX 13 KB) [file 10434_2024_16444_MOESM2_ESM.docx]

| Model | **Non-adjusted** |  |  | **Model II** |  |  | **Model III** |  |  | **Model IV** |  |
| --- | --- | --- | --- | --- | --- | --- | --- | --- | --- | --- | --- |
|  | HR (95% CI) | P value |  | HR (95% CI) | P value |  | HR (95% CI) | P value |  | HR (95% CI) | P vaule |
| OS | 0.56 (0.2~1.56) | 0.265 |  | 0.83 (0.27~2.6) | 0.754 |  | 0.74 (0.27~2.06) | 0.567 |  | 0.72 (0.25~2.07) | 0.547 |
| DFS | 1.56 (0.33~7.38) | 0.572 |  | 1.95(0.38~10.09) | 0.426 |  | 1.87 (0.39~8.92) | 0.433 |  | 2.66 (0.55~13.01) | 0.226 |

Model I：unjusted.

Model II：adjusted for age, sex, BMI, smoking and drinking.

Model III：Model II+ tumor location, operation method, type of gastrectomy, duration of surgery, blood loss and hospitalization duration.

Model IV：adjusted for all the factors.

Supplementary Table S2.Univariable and multivariable analysis of overall survival and disease-free survival in T1N1 patients
