# Supplementary material for: Is Adjuvant Therapy Necessary for Stage IB Gastric Cancer: A Retrospective Cohort Study
Source: Ann Surg Oncol. 2024 Nov 7;32(2):1210–7. doi: 10.1245/s10434-024-16444-w (PMC11698797; doi:10.1245/s10434-024-16444-w)
Supplement: Supplementary file 4 — (DOCX 12 KB) [file 10434_2024_16444_MOESM4_ESM.docx]

|  | **Total (n = 510)** | **Observation**  **(n = 271)** | **Adjuvant chemotherapy**  **(n = 239)** | **P value** |
| --- | --- | --- | --- | --- |
| **Recurrence** |  |  |  | 0.399 |
| No | 475 (93.1) | 250 (92.3) | 225 (94.1) |  |
| Yes | 35 (6.9) | 21 (7.7) | 14 (5.9) |  |

Supplementary table S4. Recurrence rate of patients in the surgery-only and adjuvant therapy groups
