# Supplementary figures and images for: Is Adjuvant Therapy Necessary for Stage IB Gastric Cancer: A Retrospective Cohort Study
Source: Ann Surg Oncol. 2024 Nov 7;32(2):1210–7. doi: 10.1245/s10434-024-16444-w (PMC11698797; doi:10.1245/s10434-024-16444-w)

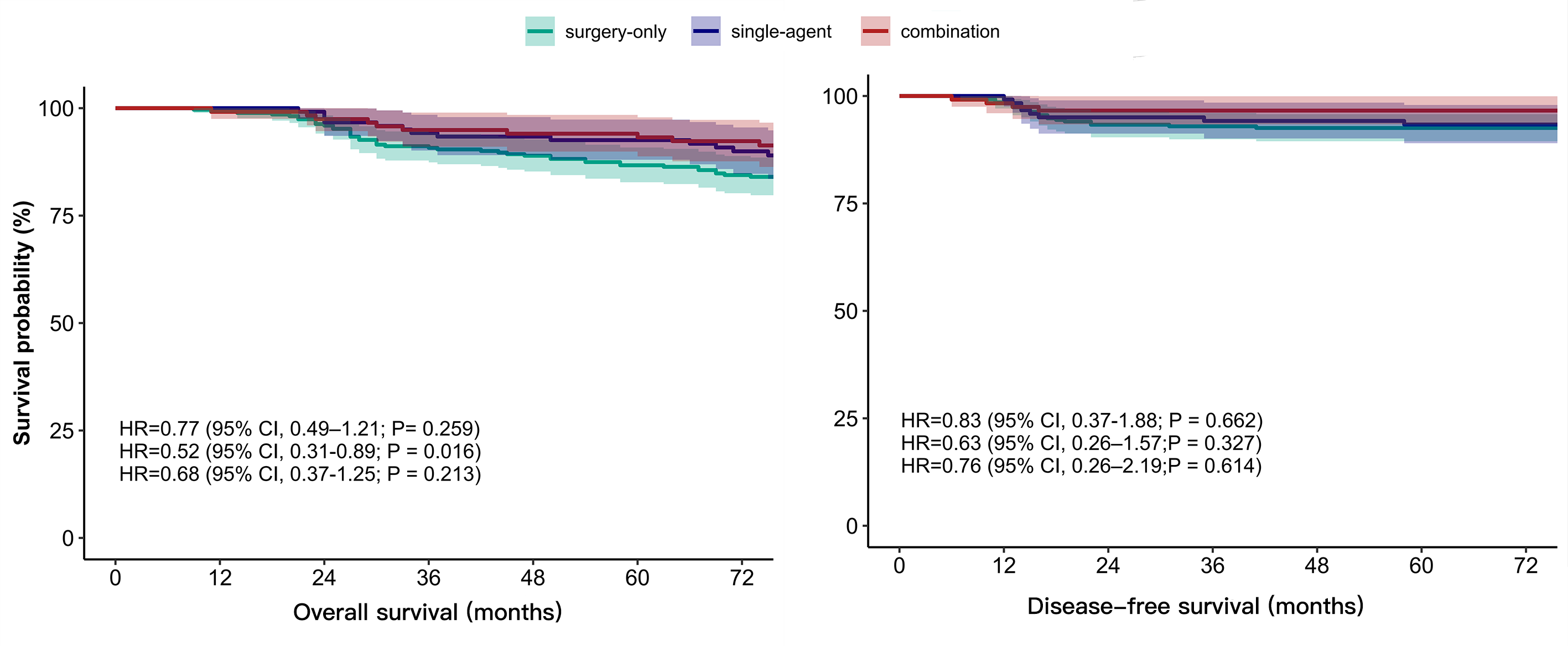

Supplement: Supplementary file 5 — (TIF 14184 KB) [file 10434_2024_16444_MOESM5_ESM.tif]

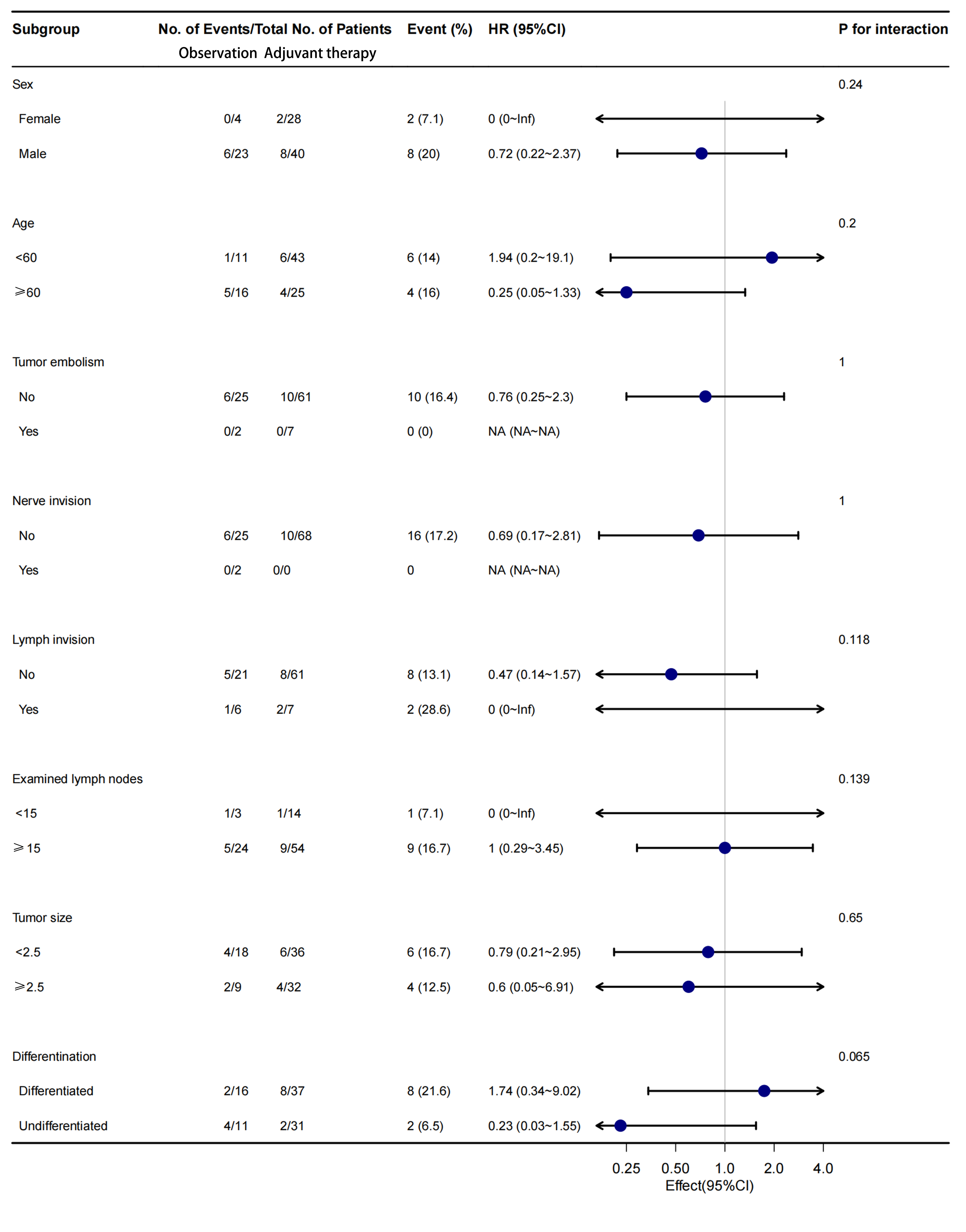

Supplement: Supplementary file 6 — (TIF 37190 KB) [file 10434_2024_16444_MOESM6_ESM.tif]

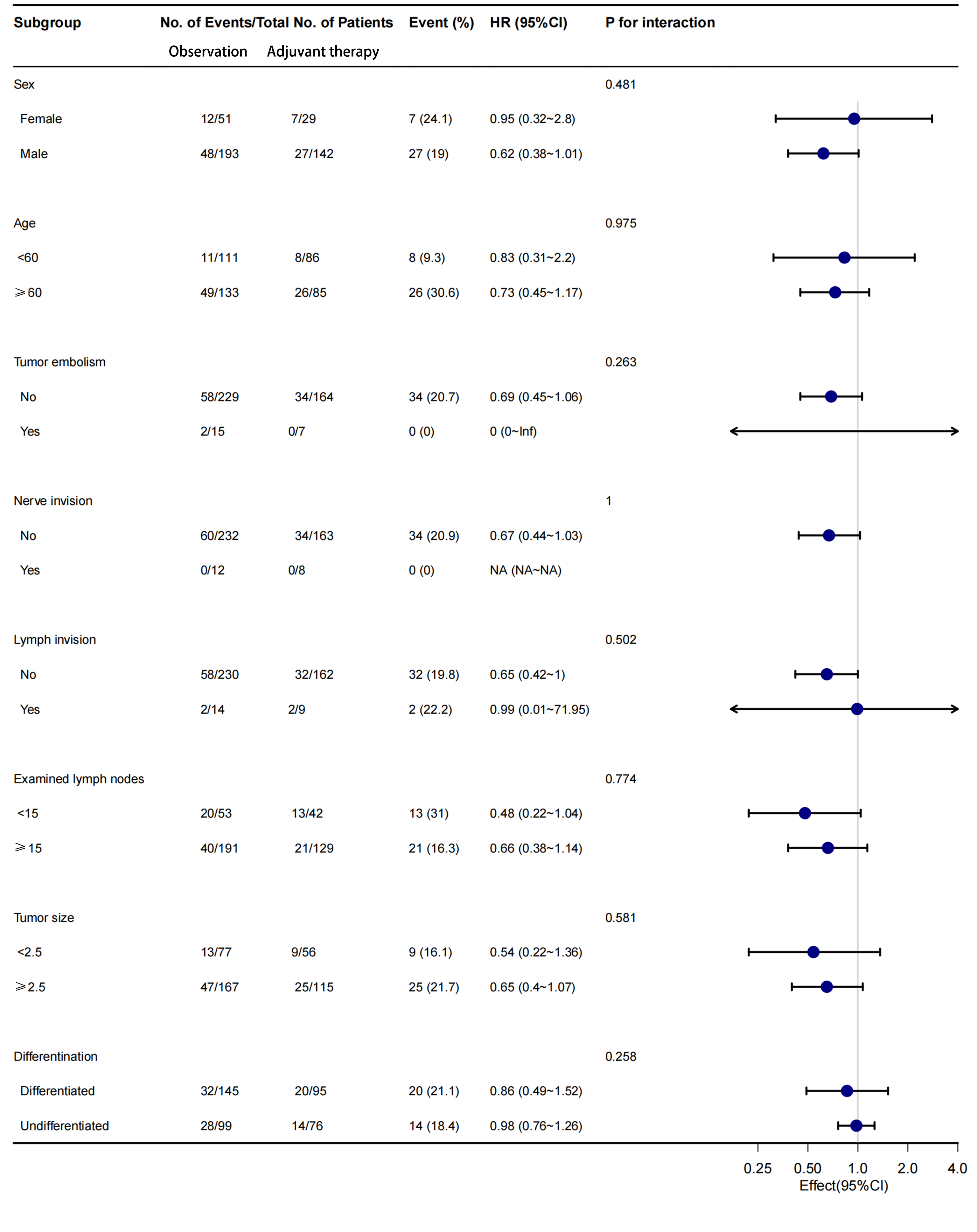

Supplement: Supplementary file 7 — (TIF 36114 KB) [file 10434_2024_16444_MOESM7_ESM.tif]
